# Boosting Confidence and Skills: A Multidisciplinary Evaluation of an Emergency Department-Based Procedure Team Rotation

**DOI:** 10.7759/cureus.77155

**Published:** 2025-01-08

**Authors:** Danielle Langan, Shorok E Hassan, Arsalan Shawl, Norman Ng, Josh Greenstein, Nehal Khamis, Barry Hahn

**Affiliations:** 1 Emergency Department, Northwell Health, Staten Island, USA; 2 Population Health, Hofstra University, Hampstead, USA; 3 Advanced Studies in Education/Master of Education for Health Professions Program, Johns Hopkins University, Baltimore, USA

**Keywords:** education, emergency medicine, procedures, resident, rotation

## Abstract

Introduction: Emergency Medicine (EM) residency programs aim to ensure residents' proficiency in performing invasive procedures. Staten Island University Hospital (SIUH) introduced an interdepartmental "Procedure Team'' to increase senior EM residents' exposure to such procedures. This study aims to evaluate the Procedure Team rotation's perceived effectiveness by assessing EM residents' improvement in comfort levels in performing procedures, EM attendings' perceptions of the curriculum's effectiveness in improving procedural and communication skills, and internal medicine (IM) resident feedback.

Methods: This is a mixed-methods study. Surveys and interviews assessed the curriculum's effectiveness, revealing feedback and areas for improvement. This study investigated the five most common procedures consulted for on the procedure team: ultrasound-guided intravenous access (US-IV), midline, central line, lumbar puncture, and paracentesis.

Results: Two hundred forty-two procedures were performed by the EM residents. On average, each resident performed 29.9 +/- 5.3 procedures per four-week rotation, with the most common procedure being the midline, which on average was performed 11.6 +/- 5.2 times, followed by paracentesis (8.9 +/- 2.1) and lumbar puncture (2.8 +/- 1.9). Response rates for the EM resident, ED attending, and IM resident surveys were 10 (100%), 23 (77%) and 27 (51%) respectively. The 10 EM postgraduate year (PGY)-3 respondents reported significant improvement in procedural comfort for midlines, lumbar punctures, and paracenteses post-rotation. Qualitative feedback from EM residents identified "education and procedural exposure" as the most valuable aspect, with 19 (70%) of 27 IM residents finding consult placement "very simple." ED-attending physicians reported increased resident autonomy over time. Suggestions for enhancing the Procedure Team included extending availability and improving communication, with 11 (41%) of IM respondents advocating for weekend coverage and a unified contact system.

Conclusions: Introducing a Procedure Team rotation in an EM residency program significantly boosts residents' confidence in performing procedures and supports interdepartmental communication and collaboration. It has the potential to enrich resident education with hands-on experience in high-risk procedures and enhance overall EM training.

## Introduction

Residents who complete a training program in emergency medicine (EM) are expected to become proficient at invasive procedures by the end of residency. The Accreditation Council for Graduate Medical Education (ACGME) sets forth guidelines for programs to follow, including a minimum number of procedures required by graduation [1]. The ACGME mandates that EM residents complete a minimum of specific procedures before graduation, such as performing 15 lumbar punctures [2]. However, procedural opportunities in the emergency department (ED) have declined in recent years due to advances in diagnostic imaging, procedural specialization, and the COVID-19 pandemic's impact on ED volumes, leaving residents with fewer hands-on experiences [3-7]. This issue is compounded as residents progress, transitioning from performing to supervising procedures.

Residency training is the primary method for instruction of clinical procedures in EM. The correlation between procedural experience and resident confidence is well-documented, emphasizing the importance of hands-on practice [8]. Unfortunately, accessing opportunities to perform certain procedures can be challenging outside of simulation or visiting rotations. While these alternatives offer value, they have inherent limitations, including cost, accessibility, and variability in real-world applicability. A recent study in internal medicine (IM) highlighted this concern, with 74.3% of housestaff reporting insufficient procedural training due to limited opportunities. Implementing a structured procedural curriculum, however, was shown to significantly enhance comfort, knowledge, and certification rates among residents [9].

To address this challenge, the ED at Staten Island University Hospital (SIUH) created a novel four-week "Procedure Team" in June 2022, designed to increase senior EM residents' exposure to invasive procedures by serving as a consulting service for the hospital. The Procedure Team aims to enhance resident education by increasing procedural experiences, promoting direct observation by EM attendings, and fostering interdepartmental collaboration. This initiative seeks to address the widespread concern of insufficient exposure to critical procedures across EM training programs for EM senior residents. Significant variability in procedural competency across EM residency programs has been highlighted, noting that residents often complete training with vastly different levels of experience in essential procedures. This disparity persists regardless of program format, hospital type, or ED volume, underscoring the need for targeted interventions to ensure consistent and comprehensive procedural training [10].

Three adult learning theories support the Procedure Team's implementation: experiential learning [11], constructivism [12], and social capital learning theory [13]. These educational frameworks underpin the Procedure Team's approach to enhancing medical education by emphasizing learning through doing, building knowledge from experience, and benefiting from multidisciplinary collaboration. 

Limited data exists discussing the potential multifactorial benefits and effects of incorporating a hospital Procedure Team led specifically by the ED. This study aims to evaluate the Procedure Team rotation's effectiveness by assessing (1) EM residents' comfort levels in performing procedures, (2) EM attendings' perceptions of the curriculum's effectiveness in improving procedural and communication skills, and (3) IM resident satisfaction and perception of this interdepartmental rotation.

## Materials and methods

This was a single-center study using a mixed methods design conducted at SIUH, a 700-bed academic tertiary care center with approximately 97,000 annual ED patient visits. The study was deemed exempt by the institutional review board (IRB# 23-0796). 

The ED Procedure Team was created as a novel educational initiative for third-year EM residents structured as a four-week rotation, designed to enhance procedural exposure and proficiency. Educational leadership noticed upon reviewing EM resident procedural logs over the past five years a deficit in certain procedures, namely paracentesis and lumbar punctures. Similar feedback from EM residents gathered through formative and summative evaluations (six-month evaluations, exit interviews with post-graduates, and annual program evaluation retreat) served as the needs assessment leading to the initiation of the ED Procedure Team. The curriculum creation process adhered to Kern’s six-step approach, focusing on the evaluation and feedback facet [14]. ED and IM leadership met to discuss the Procedure Team's practical aspects and scope as part of this interdepartmental initiative. This preparatory phase was critical for addressing logistical challenges, ensuring the rotation could integrate into existing hospital operations. The expectation was that consults placed by the IM department on weekdays before 12 PM would be completed by the Procedure Team the same day between 12 PM and 3 PM. This timing was chosen to ensure that the educational goals of the rotation were consistent with the operational demands of patient care within the hospital.

The process for placing and managing consults was facilitated via the electronic health record (EMR) system Allscripts Sunrise (Allscripts, Chicago, IL, USA). The use of an EMR allowed IM residents to identify and request necessary procedures and allowed EM residents to prioritize and perform these procedures efficiently and effectively. The EM attending physicians supervised and incorporated the Procedure Team into their observation shift responsibilities. Procedures typically performed included central venous lines, midlines, lumbar punctures, and paracenteses.

All senior postgraduate year (PGY)-3 EM residents, all IM residents, and all EM attendings who participated in the Procedure Team were eligible for inclusion. The SIUH EM residency is a three-year program with 30 residents and 10 residents per class. The SIUH IM residency is a three-year program with a total of 128 residents, 53 (41%) of which placed consults during the study period. Additionally, roughly 40 full-time attendings were employed at the SIUH ED, 30 (75%) of which were involved with the Procedure Team. The EM attending physicians' experience ranged from one to 20 years post-residency.

The authors created and reviewed the survey instrument with a consensus panel of ED medical education faculty at SIUH. This collaborative approach facilitated refinement of the survey instrument, ensuring content validity. Survey responses were stored and managed on REDCap, a secure web-based data capture application (REDCap, Nashville, TN, USA).

An anonymous single group pre-and post-test survey design was implemented to measure the change in the EM residents’ procedural comfort before and after completion of the ED Procedure Team rotation (Table 1). The survey was distributed at the beginning of the 2023 residency academic year in June and again at the end of rotation. 

**Table 1:** Emergency Medicine Resident Pre- and Post-Survey US-IV: ultrasound-guided intravenous, PGY: postgraduate year Credits: Langan, Hassan, Khamis

Section	Question	Response Options
Identifier	To match pre- and post-session surveys, please create a unique identifier in this format: Last digit of address, birth month (2-digit format), last 2 digits of phone number.	Open response field
Training Level	What is your level in training?	PGY-1
		PGY-2
		PGY-3
Gender	What is your gender?	Male
		Female
		Non-binary / third gender
		Prefer not to say
Procedure Comfort Level	What is your level of comfort performing each of the following procedures? (US-IV, Midline, Central Line, Lumbar Puncture, Paracentesis) (Scale: 1 = Not comfortable, 10 = Very comfortable)	Matrix Style (1-10)

A one-hour in-person interview with the EM residents was conducted to gather qualitative feedback on the Procedure Team's strengths and weaknesses. This session used the nominal group technique, a structured method for group discussion and idea prioritization (Table 2) [15]. The interview was conducted by a faculty member specializing in medical education and health professions, not involved in the rotation or part of ED faculty, ensuring neutrality and promoting unbiased feedback.

**Table 2:** Nominal Group Technique EM: emergency medicine, ED: emergency department Credits: Table created by authors Langan & Hassan using concepts about the Nominal Group Technique from Tran C et al., Consensus group methodology in health professions education research: the nominal group technique [15]

Step	Description
Idea Generation	EM residents were asked to write positives and areas for improvement for the ED Procedure Team on post-it notes.
Identification	The facilitator organized all post-it notes on a whiteboard, categorizing them as either “positive” or “area for improvement.”
Prioritization	Ideas (post-its) were ranked based on active participation and feedback from EM residents, with guidance from the facilitator.
Data Collection	The facilitator provided the organized and prioritized data to the research team for further analysis.

A second anonymous survey (Table 3) was designed to gather feedback from EM attending physicians on the rotation's effectiveness in enhancing procedural skills, fostering independence, and preparing residents for independent practice. The supervision versus autonomy level questions in the survey relied on the Ottawa Surgical Competency Operating Room Evaluation (O-SCORE) [16].

**Table 3:** Emergency Medicine Attending Survey US-IV: ultrasound-guided intravenous, ED: emergency department Credits: Langan, Hassan, Khamis

Section	Question	Response Options
Experience	How many years have you been practicing emergency medicine?	1-5
		5-10
		10-20
		20+
Gender	What is your gender?	Male
		Female
		Non-binary / third gender
		Prefer not to say
Observation Frequency	How many times (total) did you observe a resident physician perform each procedure during the rotation? (Procedures: US-IV, midline, central line, lumbar puncture, paracentesis)	0
		1-4
		15-9
		10-19
		20+
Supervision - Beginning of Rotation	Please choose the statement that aligns with how you felt supervising residents at the beginning of the rotation. (Procedures: US-IV, midline, central line, lumbar puncture, paracentesis)	N/A
		"I had to do"
		"I had to talk them through"
		"I had to prompt them from time to time"
		"I needed to be in the room just in case"
		"I did not need to be there"
Supervision - End of Rotation	Please choose the statement that aligns with how you felt supervising residents at the end of the rotation. (Procedures: US-IV, midline, central line, lumbar puncture, paracentesis)	N/A
		"I had to do"
		"I had to talk them through"
		"I had to prompt them from time to time"
		"I needed to be in the room just in case"
		"I did not need to be there"
Procedural Independence	The residents demonstrated the ability to perform procedures independently.	Always
		Most of the time
		About half the time
		Sometimes
		Never
Skill Improvement	The residents showed consistent improvement in procedural skills throughout the rotation.	Always
		Most of the time
		About half the time
		Sometimes
		Never
Handling Complex Cases	The residents were capable of handling complex procedural cases with minimal supervision.	Always
		Most of the time
		About half the time
		Sometimes
		Never
Communication Skills	The residents effectively communicated with patients and other team members during procedures.	Always
		Most of the time
		About half the time
		Sometimes
		Never
Rotation Effectiveness	The ED-based hospital procedure team rotation adequately prepares the residents for independent procedural practice.	Always
		Most of the time
		About half the time
		Sometimes
		Never

Finally, an anonymous survey assessing IM residents' attitudes about the Procedure Team was distributed (Table 4).

**Table 4:** Internal Medicine Resident Survey PGY: postgraduate year, ED: emergency department Credits: Langan, Hassan, Khamis

Section	Question	Response Options
Engagement	I have placed a consult or have actively taken part engaging with the ED Procedure Team.	Yes
		No
Training Level	What is your level in training?	PGY-1
		PGY-2
		PGY-3
		PGY-4
		PGY-5
Gender	What is your gender?	Male
		Female
		Non-binary / third gender
		Prefer not to say
Consult Experience	How was your experience placing a consult for the ED Procedure Team?	Scale: 1 (Very Simple) to 10 (Very Difficult)
Communication	During the Procedure Team, I was in communication with the ED team.	Always
		Often
		Sometimes
		Rarely
		Never
Relationship Improvement	The ED Procedure Team improved my relationship with the ED team.	Always
		Often
		Sometimes
		Rarely
		Never
Impact on Discharge Time	Because of the ED Procedure Team, patient time to discharge was improved.	Always
		Often
		Sometimes
		Rarely
		Never
Impact on Diagnosis Time	Because of the ED Procedure Team, patient time to diagnosis was improved.	Always
		Often
		Sometimes
		Rarely
		Never
Relevance to Practice	The ED Procedure Team was relevant to my clinical practice.	Always
		Often
		Sometimes
		Rarely
		Never
Future Improvements	How can the ED Procedure Team be improved in the future?	Open-ended response field

## Results

Data was analyzed using IBM SPSS statistics software (IBM Corp. Armonk, NY, USA). The level of significance for statistical analysis was predetermined at p < 0.05. A paired samples T-test was used to assess EM resident procedural comfort and shifts in attending physician’s procedural supervision before and after the rotation. Descriptive statistics were used to report responses from the IM resident and ED attending surveys. The results of the nominal group technique were compiled and reported providing qualitative insights into the program's effectiveness.

The five procedures studied were the most frequent consults placed by the IM team since initiation of the Procedure Team in 2022 and are essential for EM trainees to learn. These were the typical procedures done on admitted patients on the medical floors. Other procedures consulted less than twice were not included in analysis (arterial lines, Foley catheter placement, thoracentesis and ring removal). 

All 10 EM PGY-3 residents participated in the study. Of these, five (50%) were male and five (50%) were female. Table 5 demonstrates the procedural comfort of the residents before and after the Procedure Team rotation. There was a statistically significant increase in comfort level for midlines, lumbar punctures, and paracenteses.

**Table 5:** Impact of the Curriculum on Residents’ Perceived Procedural Comfort Data are presented as mean [standard deviation]. A two-tailed paired t-test was used to calculate the p-values and t-values, comparing pre-rotation and post-rotation performance for each procedure type.

Procedure Type (mean [SD])	Pre-Rotation		Post-Rotation		p-value	t-value
Ultrasound Intravenous Access	9.7	[0.7]	9.8	[0.4]	0.591	0.506
Midline	8.1	[1.7]	9.3	[1]	0.009	3.343
Central Line	9.5	[0.7]	9.6	[0.7]	0.678	0.429
Lumbar Puncture	5.6	[2.5]	8.7	[1.1]	0.007	3.493
Paracentesis	6	[2.2]	8.8	[0.8]	0.003	3.934

Data analysis from the one-hour EM residents' group interview revealed perceived areas of strength and those for improvement. The results are presented in Table 6. The most frequently mentioned strength was "education and procedural exposure," and the least frequent was "financial incentive." The EM residents did not receive monetary compensation for participating in the Procedure Team. However, some cited financial incentives as a potential benefit, referring to optimizing resource utilization, alleviating other hospital services (i.e. Interventional Radiology), and generating revenue for the ED by billing more procedures. The area for improvement most frequently mentioned was "inappropriate consults," and the least frequent was "schedule uncertainty or inconsistency."

**Table 6:** Perceived Strengths and Areas for Improvement of Procedure Team Based on Structured Interviews US-IV: ultrasound-guided intravenous

Perceived Strengths and Areas for Improvement	n (%)
Strengths	
Education and procedural exposure	15 (42%)
Good schedule	3 (8%)
Patient care	5 (14%)
Resident and attending with direct supevision	2 (6%)
Unique experience and perspectives	7 (19%)
Financial incentive	4 (11%)
Areas for Improvement	
Inappropriate Consults (i.e. US-IV)	16 (37%)
Logistics	14 (33%)
Accountability and liability/patient safety concern	2 (5%)
Interdepartmental communication	5 (12%)
Schedule uncertainty or inconsistencies	6 (14%)

Of the 23 ED-attending physicians who completed the survey, 15 (65%) were male and eight (34%) were female. The years in practice post-residency varied, with three (13%) having one to five years of experience, eight (35%) between five to 10 years, 10 (44%) between 10-20 years, and two (9%) with over 20 years of experience. Table 7 demonstrates a decrease in the level of continuous attending supervision required as the rotation progressed and more resident autonomy in performing the procedures.

**Table 7:** Paired Samples T-test for Emergency Department (ED)-Attending Levels of Supervision Data are presented as mean [standard deviation]. A two-tailed paired t-test was used to calculate the p-values and t-values, comparing pre-rotation and post-rotation levels of supervision for each procedure type.

Procedure Type (mean [SD])	Pre-Rotation		Post-Rotation		p-value	t-value
Ultrasound Intravenous Access	4.14	[2.2]	4.18	[2.2]	0.164	1.00
Midline	4.09	[2]	4.35	[2]	0.015	2.313
Central Line	2.67	[2.2]	3.00	[2.2]	0.025	2.092
Lumbar Puncture	2.87	[1.8]	3.39	[1.9]	0.007	2.642
Paracentesis	4.09	[1.3]	4.59	[1]	0.012	2.434

Of the 27 IM residents who completed the survey, 21 (77%) were male, and six (22%) were female. The participants included eight (30%) PGY-1 residents, eight (30%) PGY-2 residents, and 11 (40%) PGY-3 residents. Seventy percent of respondents indicated that it was "very simple" to place a consult. Eleven (40%) of the respondents answered the final open-ended question inviting suggestions on enhancing the ED Procedure Team's operations. Suggestions included extending availability of the Procedure Team to include weekends, improving access to contact information and schedules, and establishing a single phone number or platform for more accessible contact.

## Discussion

This study evaluated the effectiveness of a Procedure Team rotation. The findings indicated a statistically significant improvement in specific procedures, namely midlines, lumbar punctures, and paracenteses. 

This improvement may be explained by the Procedure Team's environment. Direct observation of clinical skills is a well-established tool for assessing resident procedural skills [17,18]. However, the fast-paced and unpredictable nature of the ED often makes it difficult for faculty to observe trainees directly. The Procedure Team allowed residents to perform procedures in a supervised, dedicated, and psychologically safe setting with direct feedback from an EM attending. Survey responses from EM attendings suggest that residents could perform most procedures independently by the end of the rotation, suggesting significant progression in residents’ procedural skills and autonomy.

The nominal group technique yielded substantial insights into EM resident perspectives on the Procedure Team rotation. This method highlighted the rotation's strengths, emphasizing education and procedural exposure as the most important benefit by 15 (42%) responses. Feedback from residents suggested the rotation schedule as both a strength and an area for improvement. While residents appreciated having afternoon consults on weekdays, they expressed a desire for consistency in the number of daily procedures, rotation end time, and IM resident involvement. 

The dual nature of feedback indicates that while the timing of consults was favorable, there is room to enhance the rotation's scheduling and obstacles to overcome in perfecting the Procedure Team. The highest-ranked area for improvement was inappropriate consults, with ultrasound-guided intravenous access accounting for over half of the concerns raised. Creating and distributing guidelines outlining acceptable consults to the ED and IM departments was suggested to address this challenge. Implementing such guidelines could streamline the consulting process and ensure residents are only engaged in relevant procedures. A Resident Procedure Coordinator (RPC) model has been described as a strategy to facilitate procedural consults [19]. A popular suggestion from the survey involved using a dedicated contact method (i.e. a Spectra link or Phone Number to call consults). This approach aligns with the broader literature on the advantages of establishing hospital Procedure Teams: delivering safe bedside procedures while relieving the workload on other services and reducing patient length-of-stay [20].

IM residents preferred that the Procedure Team be available 24 hours a day and on weekends to extend the accessibility of the rotation. However, this suggestion faces logistical challenges, such as the current scheduling of ED attendings as supervisors are typically on duty until 5 PM. Addressing these concerns will require new strategies to balance extended availability with supervision quality to maintain the rotation’s educational value.

This study opens several avenues for future research. Educational studies could focus on refining feedback mechanisms, exploring innovative training methods, and integrating technology to enhance procedural education. Long-term analyses may examine the sustained impact on resident performance, patient outcomes, and departmental efficiency. Notably, residents reported an improvement in hospital throughput, highlighting a potential indirect benefit to hospital operations. Future research could delve into administrative advantages, such as improved hospital metrics, financial savings, and reduced workload in other departments.

While this study primarily examined the educational benefits of the Procedure Team, future investigations could incorporate objective metrics, such as procedural success rates (e.g., first-pass success rates) and patient outcomes (e.g., complication rates or time to procedure completion). Additionally, exploring patient satisfaction scores, hospital throughput, and financial outcomes (e.g., cost savings from timely procedures) would provide a more comprehensive assessment of the Procedure Team's impact. Expanding the program to include consults from other hospital departments could further broaden its scope and maximize its benefits.

Several limitations affect the interpretation of this study. The small sample size (N=60), including 10 senior ED residents and 23 ED attending physicians, may limit generalizability. It is difficult to determine the exact number of IM residents participating in the Procedure Team at any given time, regardless of who placed the consult, which may affect representation of the feedback. A follow-up survey or group interview with IM residents could provide more comprehensive feedback.

The single-institution design also limits broader applicability, and multi-institutional research would strengthen the findings. Despite the positive feedback and observed enhanced autonomy of residents, the lack of objective measures for procedural skills introduces potential biases, particularly recall and social desirability biases, which could impact the validity of self-reported data. In this study, residents may have struggled to accurately recall specific details of their performance or experiences during Procedure Team shifts, especially given the high-stress and complex nature of these procedures. Additionally, participants may have been inclined to report higher levels of confidence or competence than they actually felt, in an effort to present themselves in a favorable light. These biases can weaken the reliability of self-reported data, potentially leading to inflated assessments of procedural confidence or perceived autonomy. To mitigate these issues, future studies could include objective performance metrics, such as direct observation or skills assessments, to validate self-reported outcomes and reduce

The absence of a control group limits the ability to attribute the observed increase in procedural confidence solely to the Procedure Team intervention. Future studies should consider multi-center designs, incorporate objective performance metrics, and include control groups to mitigate these limitations and strengthen the findings.

## Conclusions

Effective resident education and strong procedural training are essential for successful EM residency programs, where procedural proficiency is vital for patient safety. Creating a dedicated inpatient hospital rotation provides EM residents with supervised, real-time feedback on high-risk procedures. This study highlights how implementing an ED Procedure Team rotation within EM residency programs can improve procedural confidence and interdepartmental collaboration. The positive feedback from participants indicates a promising strategy for enhancing EM residency training.
